# Adaptive learning can result in a failure to profit from good conditions: implications for understanding depression

**DOI:** 10.1093/emph/eov009

**Published:** 2015-04-26

**Authors:** Pete C. Trimmer, Andrew D. Higginson, Tim W. Fawcett, John M. McNamara, Alasdair I. Houston

**Affiliations:** ^1^Modelling Animal Decisions Group, School of Biological Sciences, University of Bristol, Life Sciences Building, 24 Tyndall Avenue, Bristol BS8 1TQ, UK and ^2^Modelling Animal Decisions Group, School of Mathematics, University of Bristol, University Walk, Bristol BS8 1TW, UK

**Keywords:** behavioural shutdown model, low mood, major depressive disorder, psychic pain hypothesis, reactive depression

## Abstract

**Background and objectives:** Depression is a major medical problem diagnosed in an increasing proportion of people and for which commonly prescribed psychoactive drugs are frequently ineffective. Development of treatment options may be facilitated by an evolutionary perspective; several adaptive reasons for proneness to depression have been proposed. A common feature of many explanations is that depressive behaviour is a way to avoid costly effort where benefits are small and/or unlikely. However, this viewpoint fails to explain why low mood persists when the situation improves. We investigate whether a behavioural rule that is adapted to a stochastically changing world can cause inactivity which appears similar to the effect of depression, in that it persists after the situation has improved.

**Methodology:** We develop an adaptive learning model in which an individual has repeated choices of whether to invest costly effort that may result in a net benefit. Investing effort also provides information about the current conditions and rates of change of the conditions.

**Results:** An individual following the optimal behavioural strategy may sometimes remain inactive when conditions are favourable (i.e. when it would be better to invest effort) when it is poorly informed about the current environmental state. Initially benign conditions can predispose an individual to inactivity after a relatively brief period of negative experiences.

**Conclusions and implications:** Our approach suggests that the antecedent factors causing depressed behaviour could go much further back in an individual s history than is currently appreciated. The insights from our approach have implications for the ongoing debate about best treatment options for patients with depressive symptoms.

Pain or suffering of any kind, if long continued, causes depression and lessens the power of action, yet it is well adapted to make a creature guard against any greater or sudden evil.

               Charles Darwin

## INTRODUCTION

Depression is a state of low mood, associated with a lack of motivation, which affects millions of people worldwide [1–3]. This figure is increasing, and there is an ongoing debate about whether the condition is actually becoming more prevalent or is being over-diagnosed [4, 5]. Some authorities argue that normal sadness is increasingly diagnosed as depression, with negative consequences for the patients concerned [6, 7]. Normal sadness is triggered by external events in life. When these events are inadequately dealt with by the patient, depression may result [8, 9]. It has been argued that depression should only be diagnosed when there are no causal external factors [7] but support for this is mixed [10]. For example, it is generally agreed that depression should not be diagnosed in cases of bereavement, but some have argued that this exclusion should be extended to other causal factors of sadness [11]. The difficulty is in assessing what factors should be taken to be causal. In this article, we focus on healthy reactive behaviour (which might easily be interpreted as ‘depression’), rather than mental disorders. Although we describe the two as distinct, it is possible that the effect of strong reactions may increase the likelihood of more permanent changes in brain function (perhaps resulting in depression as a mental disorder). We do not address that possibility in this article but focus on why reactive effects can persist for a long time. Two recent meta-analyses have shown that pharmacological treatments are effective only in the most severe cases of depression [12, 13] so it is clear that preventing and curing depression require a deeper understanding of how it arises and persists.

Evolutionary psychiatry has provided several explanations for depression based on the view that it is an adaptive response to a problem [14–17] (see Coyne [18] for a critique); this is an approach that has been applied to other medical issues [19]. Common to many of these explanations is the view that emotions, and their associated disorders, have evolved as mechanisms for guiding adaptive behaviour [20, 21]. The behavioural shutdown hypothesis of depression [22, 23] supposes that individuals should become inactive if cues from the environment indicate that activity would decrease their Darwinian fitness. The variability of environmental conditions faced by most organisms—including ancestral humans—means that it will sometimes pay to be active, such as when food is abundant, and sometimes pay to be inactive, such as when food is scarce or when there is danger [24]. Depression would then be an appropriate response to the current conditions if the affective experience acts as a mechanism for implementing appropriate behaviour.

Many theories which explain depression as an adaptive response to environmental conditions fail to explain why low mood persists when conditions improve. Individuals typically do not have perfect knowledge about current conditions, because conditions change over time and outcomes are stochastic. Faced with this uncertainty, animals will have evolved psychological mechanisms to learn about the conditions and respond appropriately given their current information [25–27]. We shall assume that evolution has generated cognitive and decision-making processes that adjust behaviour optimally in response to data received about the environmental conditions. Despite an individual following the optimal strategy (producing the best possible behaviour, given the data available), an external observer who knows the conditions perfectly (hereafter referred to simply as an ‘omniscient observer’) may witness seemingly maladaptive behaviour [28, 29]. It would be easy for such an observer to infer a psychological disorder, even though the strategy is optimal. Inactivity under good conditions, which is not in itself beneficial and may appear similar to some forms of depression, may therefore arise from an optimal mechanism. This is the angle that we explore in this article.

To study how such seemingly maladaptive behaviour may arise from optimal decisions in a variable and uncertain environment, we consider a simple scenario in which environmental conditions are either ‘good’ (where rewards are likely to be obtained when the individual is active) or ‘bad’ (where rewards are less likely) and can switch between those states over time. At any time, the switching rate is either fast or slow. The switching rate can itself also occasionally switch from one rate to the other. Although this is a crude simplification of the pattern of change in real environments, it serves as a convenient way to illustrate some general principles. We assume that natural selection has resulted in individuals that are adapted to the probabilities of environmental change, so that they learn optimally about the probable state of the world from the outcomes of their actions. We find that the optimal strategy for maximizing reward rate can result in a significant minority of individuals failing to take advantage of good conditions.

## THE MODEL

We consider an individual whose behavioural strategy has evolved to maximize its long-term rate of reward (e.g. net rate of energy gain, [30]). The individual makes repeated decisions of whether to expend effort (hereafter ‘trying’) or be inactive (hereafter ‘resting’) for one time step. Resting is assumed to have a payoff of zero. Trying will result in success or failure. If the outcome is failure, the individual receives a negative payoff, *Z*, reflecting the costs of expending effort (e.g. the extra metabolic cost of foraging). If the outcome is success, the individual receives a positive payoff, *X*, reflecting the net benefit of the reward (e.g. the energy of a food item minus the extra metabolic cost of foraging).

**Figure 1.**
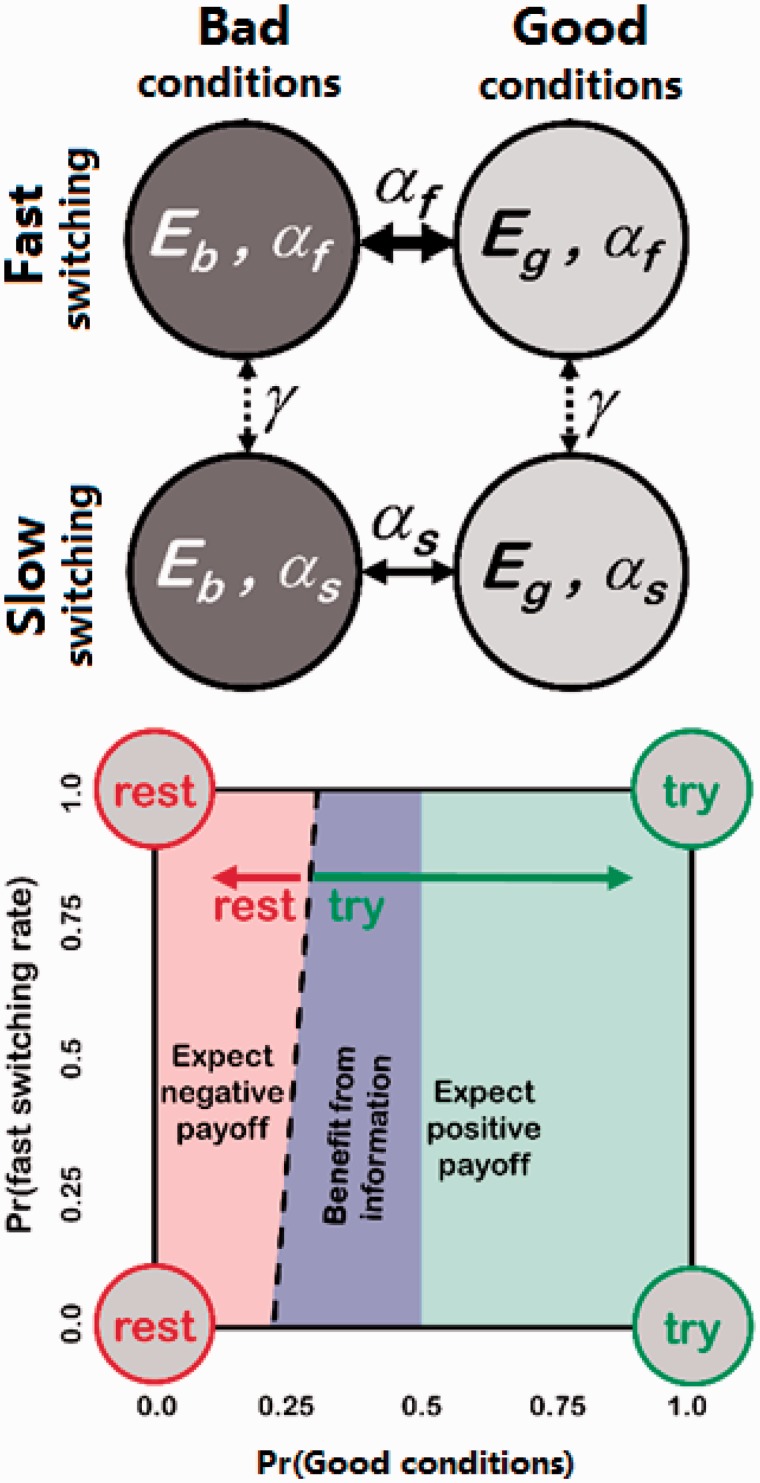
(**a**) The four possible environmental situations at any given time. In the fast-switching case (*α*_f_), the environmental state changes between high (good, *E*_g_) and low (bad, *E*_b_) reward probabilities frequently (thick solid arrow). With a low switching probability (*α*_s_), the environmental state tends to remain good or bad for a long time (thin solid arrow). With a very small probability *γ*, the switching rate can change between fast and slow (dashed arrows). (**b**) Approximate form of the optimal strategy, to aid intuition. The individual’s belief about the current environmental situation is three-dimensional, which is difficult to visualize. Here, we represent belief state in two-dimensions, as though we had summed across the joint probabilities to obtain the most common decision with respect to each combination of *P*(*E*_g_) and *P*(*α*_f_). The circles at each corner show the appropriate behaviour if knowledge were perfect (i.e. each probability is either 0 or 1). The individual should try (green shading) if the probability of good conditions (i.e. a high reward probability) is large and rest (red shading) if it is small. Due to the need to learn about current conditions, there is a range of belief space where the individual should try even if the probability of a reward is low (purple shading). The boundary between trying and resting (dashed line) is influenced by the probability that the switching rate is fast, because this alters the value of information

We assume that the probability of success when trying depends on the current environmental conditions; it takes one of two values, *E*_g_ or *E*_b_ (with *E*_g_ > *E*_b_, representing good and bad environmental states, respectively), but will sometimes change between them. The probability that the environmental conditions change from good to bad or *vice versa* in a given time step (hereafter the ‘switching probability’) is either *α*_f_ or *α*_s_, with *α*_f_ > *α*_s_ such that *α*_f_ (fast switching) implies a more changeable environment than *α*_s_ (slow switching). Figure 1 shows the four possible environmental situations: good or bad conditions, with either a fast or slow switching rate. We assume that evolution has resulted in individuals behaving as if they know the values of *E*_g_, *E*_b_, *α*_s_ and *α*_f_ but do not know which of the four combinations of those values (see Fig. 1) holds at any given moment. There is a meta-probability, *γ*, that the switching probability changes in a given time step, but its value is very small, such that the switching probability is highly unlikely to change during a time step. We assume that behaviour is evolutionarily adapted to this meta-probability.

Initially the individual has no knowledge of the current probability of success (*E*_g_ or *E*_b_) nor the current switching probability (*α*_f_ or *α*_s_). To maximize its long-term rate of reward, the individual learns about the current values and uses this information to decide whether to rest or try at any given moment. Trying, and getting a success or a failure, will provide information about which of the probabilities of success is most likely. Repeated trying will also inform the individual about the current switching probability. At any given moment, we can summarize the individual’s knowledge about the environment in terms of three joint probabilities, *P*(*E*_g_, *α*_f_), *P*(*E*_g_, *α*_s_), *P*(*E*_b_, *α*_f_), from which the fourth probability can be calculated, as the four joint probabilities must sum to 1. The position of an individual in this belief space is updated in a Bayesian manner (see Supplementary Appendix). Using dynamic programming [31, 32], we can then find the optimal strategy that specifies whether it is better to rest or try given the current estimates. The optimal strategy will try when the environmental conditions are likely to be good, but it may also be best to try when conditions are relatively uncertain, to gain information for future time steps (Fig. 1b). A full technical description of the model is provided in the Supplementary Appendix.

We then track the trajectory of individuals following the optimal strategy through this probability space as they make decisions and learn about their environment. We use the same set of parameter values throughout (*E*_g_ = 0.8, *E*_b_ = 0.2, *α*_f_ = 0.4, *α*_s_ = 0.01, *X* = 1, *Z = *−1, γ = 0.001).Note, however, that the insights gleaned from the model are not dependent on any particular set of parameter values. With *Z* = −*X* (as we assume) it is important that the probability of success is sometimes >0.5, because it is then worth trying, and sometimes <0.5, because (from the perspective of someone who knows the conditions) it is then better to rest. However, since the individual does not have perfect knowledge, it should sometimes try even if success is less likely than failure, because the individual gains information by trying. By using probabilities of success of 0.8 in the good environment and 0.2 in the bad environment, we ensure that an individual can, after only a few attempts, gain a significant amount of information about which environment it is in. With such different probabilities of success, it is also important that the individual makes the correct choice.

## CHARACTERIZING DEPRESSION

Numerous definitions of depression have been proposed, reflecting the wide range of different perspectives on this multifaceted phenomenon. Nevertheless, as Gilbert [1, p.143] says,

depression has some built-in pattern; it is a potential brain state organization and any of us are potential sufferers. It will have great phenotypic variability from person to person but still there will be commonalities; an experience of misery, low levels of explorative behaviour, loss of energy, negative self-organizations and perceptions of inferiority and poor assertiveness.

Depression can vary in magnitude from mild to very severe [33]. In the most severe cases, it seems clear that the phenomenon is a form of illness. In less severe cases, it is not clear whether depressed experiences and behaviour are caused by a malfunction of the brain or the reaction of a healthy brain to the set of experiences. In this article, we focus on the latter possibility, of depressive behaviour being a reactive occurrence in a healthy individual.

In affective terms, depression is widely recognized as a persistent state of low mood, involving a suite of correlated changes in mental processes affecting motivation and cognition [34]. The mental processes govern the behaviour of the individual, and it is that resultant behaviour which is subject to natural selection. We therefore focus on resultant behaviour rather than the mental processes that generate that behaviour, on the assumption that evolution has produced mental processes that implement the optimal strategy.

Even from a behavioural perspective, and within the framework of this simple model (where we are concerned purely with behaviour; i.e. the explorative option of trying), it is difficult to define depression in a satisfactory manner. In part, this is because depression is regarded in different ways; this article deals with different meanings, each of which has different problems, as identified later.

1. It would be easy to characterize depression as resting when it would be better to try (i.e. resting despite success being more likely than failure). However, because the environment can switch type, it would be strange to regard an individual that had experienced repeated failures in the bad environment as depressed simply because the environment had only just switched to the good environment.
2. It could be argued that depression relates not to a single decision but to a series of decisions. In this case, the number of time steps of not trying (in either environment) could be used as a measure of depression. However, from an omniscient perspective, resting is the optimal behaviour in the bad environment, so would be unlikely to be regarded as maladaptive (or labelled as depression) by others.
3. As a refinement, the number of time steps of resting whilst in the good environment could be used as a measure of depression. However, because such behaviour is adaptive in the bad environment, the possibility of the environment switching types makes such a definition questionable.
4. As an extension to (3), it could be imposed that the individual has already tried (at least once) since entering the good environment. But as they may then have just been unlucky and had an experience which happened to be bad, it would again seem dubious to call such behaviour ‘depressed’.
5. To avoid the earlier problems, it makes sense to define depression (from a behavioural perspective) as something that can only be identified in the good environment, and where the individual has already experienced success since the environment has become good. Our most stringent definition, therefore, is to characterize depression as resting when in the good environment (i.e. when it would be better to try), despite having tried and succeeded since having entered the good environment.

This final, most stringent, definition captures two key features of interest. First, from the perspective of an omniscient observer, the depressive behaviour itself is not beneficial, since in good conditions it is always better to try than to rest. Second, the individual behaves as though conditions are bad despite recent evidence to the contrary—namely a success, which always increases the probability that conditions are good. Later, we show that the stringent definition of depression can be met even when individuals follow the optimal strategy.

## RESULTS

**Figure 2.**
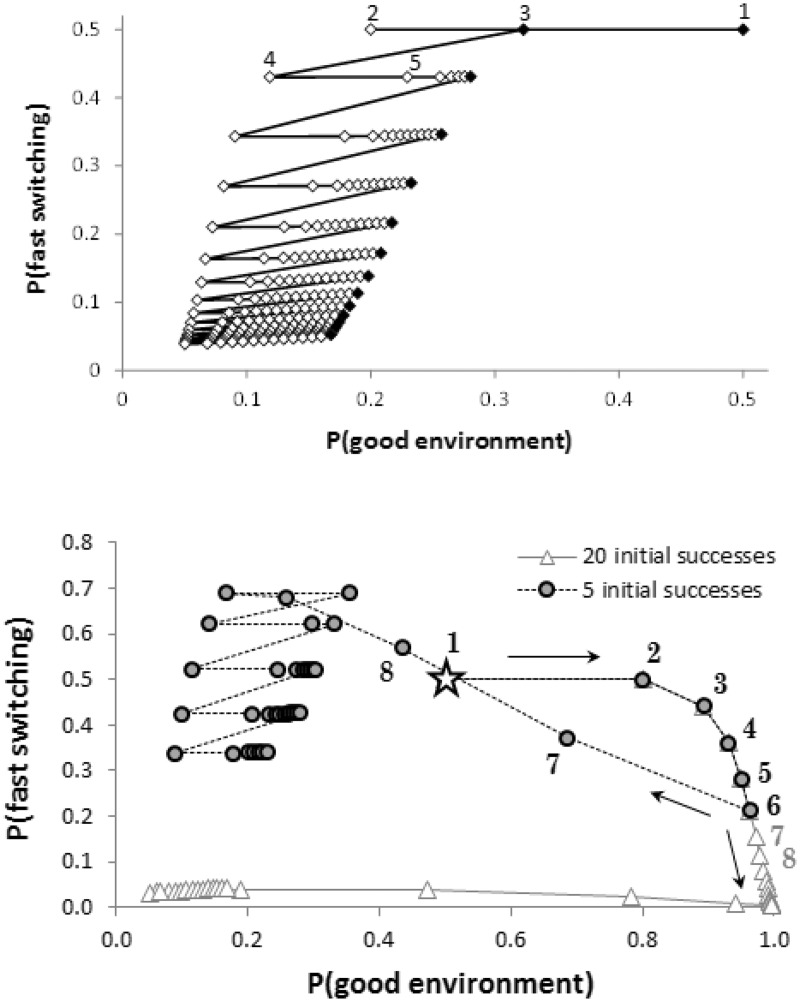
(**a**) Effect of a sequence of failures on the two probabilities P(*E*_g_) and P(*α*_f_) for an individual following the optimal strategy, where the outcome of trying is fixed as a failure. Black markers indicate trying whereas white markers indicate resting. The values on each axis are each a sum of probabilities; at each data point, *P*(*E*_g_) = *P*(*E*_g_, *α*_f_) *P*(*E*_g_, *α*_s_) and *P*(*α*_f_) = *P*(*E*_g_, *α*_f_) + *P*(*E*_b_, *α*_f_). (**b**) The effect of a sequence of failures depends strongly on how many successes were previously experienced. The figure shows two sets of trajectories, each for 40 time steps, each starting at P(*E*_g_) = 0.5, P(*α*_f_) = 0.5 (indicated by the star symbol). The solid grey line (triangle markers) corresponds to 20 successes followed by 20 failures. The dashed black line (circle markers) shows five successes (following the exact same path for those time steps) followed by 35 failures. Following 20 successes, it only takes a few failures for the individual to decide to rest for a prolonged period (appearing ‘depressed’ by Characterization 2), because it is relatively sure that current conditions are bad and that the switching rate is slow. However, if the environment has just switched from bad to good, the resultant behaviour would be regarded as depressed under Characterization 3 and, with one unlucky outcome, even Characterization 4, as the individual would then be choosing to rest despite being in the good environment

We assume that the individual starts with no information about the current state of the environment and thus all four states are equally likely (i.e. P(*E*_g_) = 0.5, P(*α*_f_) = 0.5). The decision to rest (rather than try) for numerous time steps can be induced by a sequence of failures (Fig. 2a). Following the first decision, the perceived probability that trying is likely to be rewarded declines because the individual experiences failure. Nothing is learned about the switching rate on this first update, but subsequent failures increase the perceived probability that the switching rate is low. After the first try, the individual sometimes chooses to rest. During these periods of resting, P(*E*_g_) increases due to the possibility of conditions having switched during that period. P(*α*_f_) alters very little when resting, due to the small meta-switching probability, *γ*. Note that as the length of the sequence of failures increases, there is an increase in the duration of resting before the individual tries again; these numerous time steps of not trying would be regarded as increasing degrees of depression under Characterization 2.

Following a string of successes, the individual will keep trying because, with each success, the perceived probability of reward increases further. Thus, the individual will only stop trying if it experiences failures. A long sequence of successes followed by a few failures can lead to very long periods of resting, because the initial string of successes leads the individual to believe that the environment switches only rarely (and the latest data suggest that conditions are bad). By contrast, a short sequence of successes followed by a failure indicates that the environment switches frequently and so the periods of resting will be short (Fig. 2b). Contrasting the estimates after 40 time steps for the two cases in Fig. 2b, we see that the history of experiences can have a long-lasting effect on behaviour (for considerably >20 time steps).

**Figure 3.**
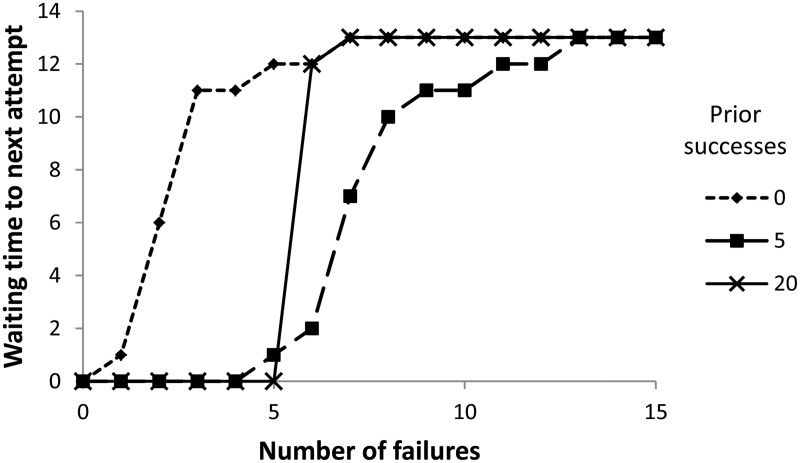
The effect of the number of consecutive failures on the waiting time to the next attempt, depending on the number of previous successes (0, 5 or 20) experienced initially by a naïve individual

In Fig. 3, we show how the duration of resting increases with the number of consecutive failures (i.e. unsuccessful tries) that have been experienced, and how this depends on the number of consecutive successes experienced previously. Note that in each case the duration of resting asymptotes because, even though conditions may initially have been bad, the conditions will eventually become good (as determined by the switching probability).

Counter-intuitively, the response to repeated failure shown in Fig. 3 following 20 prior successes falls in between that following 0 prior successes and 5 prior successes. This result is caused by learning about the environmental switching probability. In both the 0 and 20 cases, there is good reason after six failures to think not only that the environment is currently bad, but that the switching probability is low. With five successes followed by some failures, there is more reason to believe that there is a high switching probability, meaning that more failures are required before the individual waits a long time between attempts. Thus, individuals that were previously used to good conditions for a long time become more thoroughly depressed (according to length of time not trying) after a sequence of failures.

In the long term (i.e. after 1000 time steps, by which time the system has settled down to stable values), the percentage of individuals that are trying in each of the four environmental situations is as follows: 68% under good, fast-switching conditions; 91% under good, slow-switching conditions; 61% under bad, fast-switching conditions and 18% under bad, slow-switching conditions. Thus, even when exposed to good conditions that are unlikely to change soon (*E*_g_, *α*_s_), almost one in 10 individuals are inactive. Under Characterization 1, such individuals (and the 30% not trying under good, fast-switching conditions) would be regarded as depressed.

**Figure 4.**
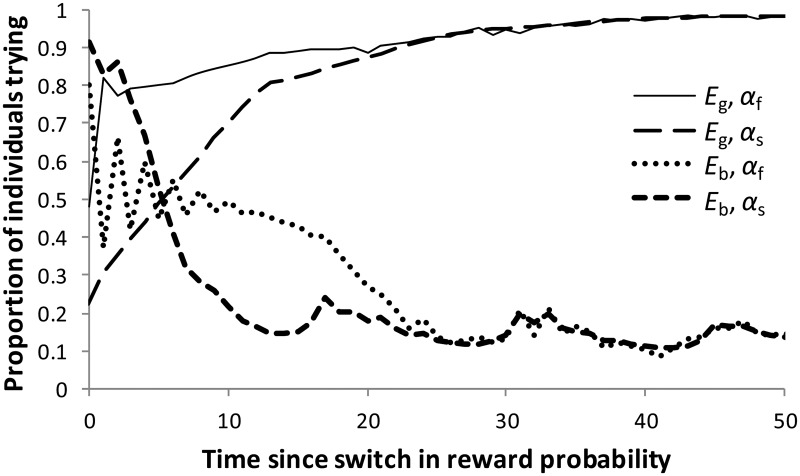
The proportion of individuals trying in each environmental situation with respect to the time since the last switch of reward probabilities (*E*_g_ to *E*_b_ or *vice versa*). These proportions were calculated from 100 000 individuals each experiencing 2000 time steps

If good conditions (*E*_g_) persist for an extended period, the percentage of individuals that try tends to 100%. This takes some time so that even 20 time steps after conditions have switched from bad to good, and there is a significant proportion of individuals that are inactive. However, the majority of individuals who are inactive when conditions are good have switched to those conditions relatively recently, as shown in Fig. 4.

## FORCED ATTEMPTS

The results so far have assumed that each individual has a free choice of whether to try or rest at each time step. However, in the real world, individuals are often ‘forced’ to experience the current environmental conditions, either through necessity (e.g. the need to find food to avoid starvation) or because they are coerced or encouraged (or, more simply, informed) by others. We therefore incorporated a fixed probability of being forced to try in any given time step, and allowed the strategy to take this probability into account (see Supplementary Appendix). This allows us to consider the effect of forced attempts on the optimal behavioural choices of when to try.

**Figure 5.**
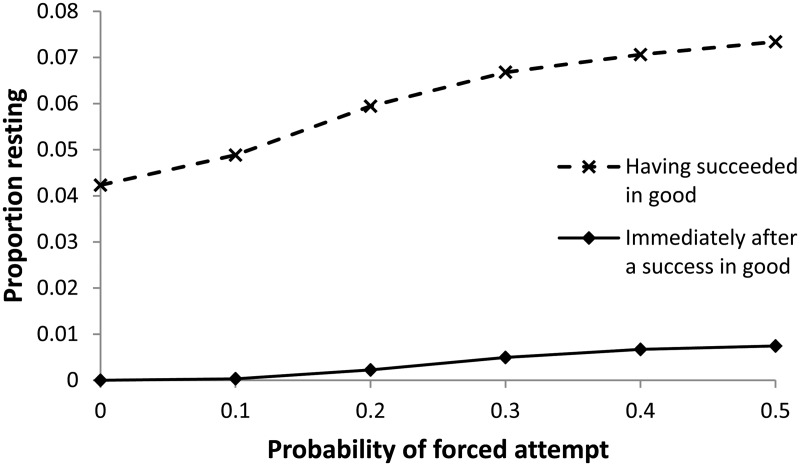
The proportion of individuals under good conditions that choose to rest (when they have that choice) increases with the probability of forced attempts, both immediately following a success (solid line) and having already tried and succeeded at some point since conditions became good (dashed line)

**Figure 6.**
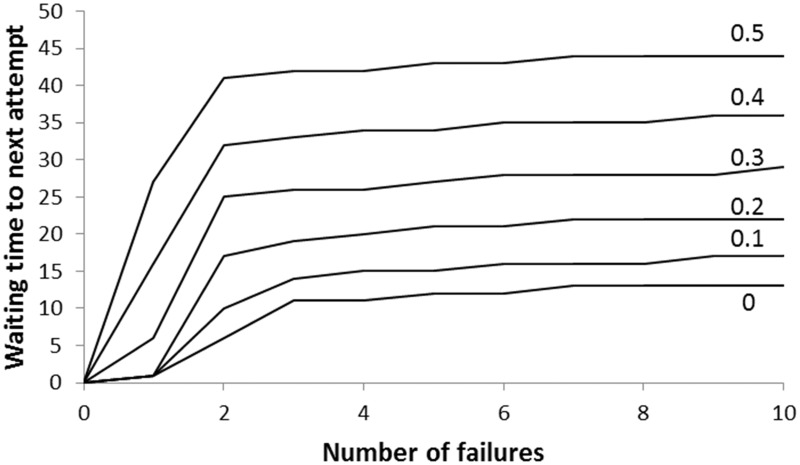
The anticipated probability of forced attempts (labels on lines) affects the waiting time to the next attempt when those forced attempts do not occur. In the baseline case of no forced attempts, the asymptotic waiting time with respect to the number of failures is 13 resting periods; for a 50% probability of forced attempts this increases to 44 consecutive decisions to rest (i.e. much more depressed according to Characterization 2)

Without forced attempts, individuals should always try (again) following a success. This is because an attempt should only have been made if the individual’s belief that conditions were good, P(*E*_g_), was sufficiently high to put it in the region of state space where trying is the better option (see Fig. 1b) and a success increases this probability. However, with forced attempts, it is possible for an individual to experience a failure despite being within the region of state space where it would have been better to rest, and the increase in P(*E*_g_) following a subsequent forced attempt which is successful may not be sufficient to move it into the region where it should try at the next opportunity. The solid line in Fig. 5 shows the effect of forced attempts on the proportion of individuals that are inactive under good conditions, immediately after experiencing a success. Anticipation of forced attempts can also make individuals more reluctant to try through their own volition (Fig. 6). This is because when forced attempts are anticipated, the informational benefit of trying is reduced, so the individual should be less inclined to try when conditions are believed to be bad (the information gain zone in Fig. 1b is smaller). This greater reluctance to try leads to longer periods of inactivity when forced attempts do not occur (i.e. the anticipated additional data do not arrive).

It is possible that, having switched to good conditions and experienced success, a subsequent run of bad luck causes an individual to stop trying; this is shown as the dashed line in Fig. 5. Both mechanisms—a forced attempt, or success followed by bad luck—result in behaviour that, according to our most stringent Characterization 5, can be characterized as depression: a small fraction of individuals show inactivity when environmental conditions are good, despite having experienced success since environmental conditions became good.

## DISCUSSION

We have used a simple model to show that seemingly depressed behaviour can arise from an optimal strategy when the individual has had an unfortunate series of experiences, and that the antecedent factors causing this reactive behaviour could go a long way back in an individual’s history.

Depression has usually been viewed as a mental disorder that is maladaptive [35]. However, there have been a number of recent suggestions that depression represents an adaptive response to complex problems. For example, the analytical rumination hypothesis and the social navigation hypothesis suggest that depression aids rumination on difficult social problems and/or influences others to do a greater share of required work [14, 17, 36–38]; the social risk hypothesis proposes that depression can minimize the risk of social exclusion by reducing an individual’s propensity to engage in risky behaviours [39, 40]; and the infection-defence hypothesis proposes that an individual who is susceptible to illness can reduce the risk if they remove themselves from company [41, 42], which goes some way to explaining why depression is, in many ways, similar to sickness behaviour. Despite providing possible insights into aspects of depression, these adaptive hypotheses do not account for why low mood can persist in good conditions.

Rather than arguing that depression is an adaptive phenomenon, we take a more nuanced view. Our model identifies when it is optimal to act, and when to refrain from taking action. However, the depressive behaviour that we predict in a small fraction of individuals following this optimal strategy is not, in itself, beneficial—indeed, our most stringent definition only regards inactivity as depression if it would actually be better for the individual to be active in those circumstances, and then only if the individual has gained recent evidence that conditions are favourable. In short, the optimal strategy we have identified is adaptive, but the depressed behaviour it sometimes produces is detrimental (from an omniscient perspective). Our model does not assume that payoffs are necessarily social or immunological and suggests that mechanisms supporting such behaviour may also evolve in non-social contexts.

Our results are relevant to learned helplessness [43], in which repeated failures can lead to individuals no longer trying to solve a problem. Seligman noted the great similarity between depression and learned helplessness, for instance comparing impairment of behaviour in naturally occurring depression with induced learned helplessness in laboratory settings [44]. Because our analysis is from an entirely behavioural perspective, it could be said that our results relate more strongly to learned helplessness than to low mood or depression, which are unobservable psychological states that potentially underlie such behaviours. Learned helplessness is often considered in the context of the ability of an individual to control their environment through their choice of actions [43], though Teodorescu and Erev [45] suggest that rather than learned helplessness correlating with perceived controllability of the environment, reward prevalence (i.e. frequency of reward) governs exploration. In our model, the individual never has control over their environment—only their behaviour, of whether to engage with the environment—so the paradigm is subtly different to the classical view of learned helplessness. Whereas learned helplessness is most often associated with failure to avoid costs (such as pain) when they could be avoided, our approach shows why, from a theoretical perspective, individuals can learn not to try for benefits (such as food) even when they are available.

Keller and Miller [46] point out that severe depression can be profoundly harmful; so much so that the behaviour is clearly not adaptive, for instance in the case of suicide [18]. Although we have not considered such cases in this article, we have two points to make on this topic. The first is that, as identified earlier, many articles on evolutionary psychiatry present depression as adaptive in and of itself, whereas we highlight that even when the behaviour in question is not beneficial from the perspective of an omniscient observer (in this case, witnessing the individual resting when the environment is good), it can have come about through using a strategy which is evolutionarily adaptive. Second, we have not dealt with cases where the strategy is non-adaptive (as is surely sometimes the case). Nevertheless, our model indicates that seemingly maladaptive behaviour can often be understood from an adaptive perspective by looking further back into an individual’s history to antecedent factors. This insight has implications for the ongoing debate about treatment options for patients with depressive symptoms. Kendler and Gardner [47] highlight the difficulty of inferring causal pathways for the occurrence of depression; by showing that antecedent factors may have occurred a long time ago, our results encourage a similar conclusion.

As well as the differing probabilities of reward, our model includes two possible environmental switching rates between those reward conditions. This somewhat unusual assumption is crucial to some of our results, because an individual then learns not only about the current reward probability but also about how long the reward conditions are likely to persist. One might typically expect that a small number of failures should not lead to depressive behaviour. One of the striking results of this model is that optimal behaviour dictates that a sufficiently long sequence of successes can then result in ‘giving up’ behaviour after only a small number of failures, due to the original sequence having provided evidence that the environmental conditions do not often change. An important assumption behind this result is that a switch from good to bad conditions has the same probability of occurrence as a switch from bad to good conditions. If these probabilities were not symmetrical, then initial exposure to good conditions might instead tell the individual that the world is a generally favourable place, so the individual would therefore be less likely to give up after a short sequence of failures. In other words, the assumptions about changes in environmental conditions can have significant effects on the class of behaviours which emerge from the modelling.

As well as relating to depression, or low mood state, our results are directly comparable with the partial reinforcement extinction effect (PREE) [48, 49], in which animals that have only occasionally been rewarded for a particular action continue to take that action for longer (in the face of repeated failures) than if the action had previously been rewarded consistently. In our model, if an individual has experienced repeated successes, then after a few consecutive failures the decision will be made to rest for a considerable period. After more mixed results of successes and failures followed by constant failure, an individual will continue to try for a longer period. Thus this model does, to some extent, account for the PREE [50, 51]. Recent work on the PREE has identified that the effect may be governed by how attractive the options are relative to each other; this effect can be captured in a model by assuming that individuals using only very recent data [52]. It would be interesting to extend our model to allow payoffs to differ in magnitude to determine whether the Bayesian approach produces similar behaviour.

Huston *et al.* [53, p.249] identify that ‘the withholding of expected rewards results in extinction of behaviour and, hypothetically, to depression-like symptoms.’ This view accords with our model and Huston *et al.* show that, in rats, anti-depressants reduce the tendency to display extinction-induced withdrawal. The expected benefits (and costs) of using anti-depressants will depend on circumstances, so their use can be debatable [54].

Signal detection theory [55] tells us that individuals must consider the expected costs and benefits associated with each option to consistently choose the best action. Nesse [56, 57] uses signal detection theory to identify why many defensive actions might be expressed more readily, and more intensely, than might otherwise appear to be optimal. If the cost of mounting an unnecessary defence is small, and the consequences of not mounting a necessary defence are large, then the optimal setting will often result in a tendency towards defensive action that might, to an omniscient observer, seem overcautious. Nesse argues that this ‘smoke-detector principle’ can explain a multitude of defensive actions, such as anxiety. Our results also relate to this principle, because optimal decision making must depend on the expected cost of unnecessarily trying (when in the bad environment) and the rewards that are being missed when not trying in the good environment. In our model, the immediate payoffs for correct or incorrect behaviour must be combined with the longer-term benefit of information acquisition from trying (Fig. 1b). Although this explore–exploit trade-off tips the balance towards trying when good and bad environments are equally likely, it also means that when not trying, the individual’s information state alters very little; this can result in periods of not trying despite being in the good environment.

For simplicity, our model assumes that each individual maximizes their long-term rate of reward, but in many contexts, there are other things to consider. For instance, with respect to energy levels, an animal will also need to take more immediate factors, such as the risk of starvation, into account. In terms of energy, this model can be regarded as a proxy for maximizing reproductive success in a situation where the individual’s reserves are not close to zero or their maximum value [31]. Models that include energy reserves as a state variable could produce similar effects to those we have found (e.g. Nettle [58] does this, though in a form which assumes the effect rather than showing it). By focusing on a simple model based on rate of gain, we have shown that reserve-dependent behaviour is not required in order for optimal decisions to meet a stringent criterion for depression. Our simplifying assumptions—that the environment is either good or bad at any one time and that the switching rate is either fast or slow, rather than taking a value from a continuous range—certainly do not hold in the real world. However, these assumptions enable the logic behind the results to be illustrated clearly and allow tractable calculations of optimal behaviour, which would not be possible in a much more complicated model.

Our model is not species-specific, which suggests that more work on models of depression in non-human animals may be instructive [59, 60]. However, as models become more specific (e.g. focusing on social competition), great care will be required in extrapolating conclusions from other species to humans. For instance, in rodents, the usual depression mechanism of mammals may have been adapted to result in hibernation [61].

The Diagnostic and Statistical Manual of Mental Disorders (DSM) distinguishes between major depressive disorders and ‘normal’ sadness. One recent debate has focussed on the distinction between depression and normal grieving. DSM-IV included a ‘bereavement exclusion’, suggesting that people should not be diagnosed as having a depressive disorder within a couple of months of bereavement. However, the most recent version of the DSM (5) has removed this exclusion. The model presented here is not suitable for addressing the issue of bereavement exclusion because the only costs are small ones of trying (when it would be better to rest) rather than high magnitude losses such as bereavement. However, it may be possible to build similar models that incorporate loss magnitude to help address the question of whether bereavement should exclude the diagnosis of a depressive disorder.

From a clinical perspective, there are many forms and characterizations of depression, with psychomotor activity being most reduced in the case of melancholic depression [62]. Our approach has taken the simplistic view of characterizing depressive behaviour as inactivity when it would be better to try. However, our model only relates to the reactive phenomena characteristic of non-melancholic forms of depression, which are the more common [63]. Consequently, our model does not account for the reduced psychomotor activity of melancholic (or endogenous) forms of depression.

In this article, we have identified a strategy for maximizing the reward rate when an individual’s only control over the situation is deciding which option to take. A somewhat deeper sense of the term ‘depression’ may relate not so much to the immediate action as to one’s perceived ability to control subsequent (currently unknown) situations, leading depressed individuals to avoid situations which are less controllable (e.g. social situations). We believe that understanding this issue, which may be fundamental to developing more effective treatments, will be greatly assisted by taking an evolutionary approach.

## Supplementary Material

Supplementary Data
