# Supplementary material for: Adaptive learning can result in a failure to profit from good conditions: implications for understanding depression
Source: Evol Med Public Health. 2015 Apr 26;2015(1):123–35. doi: 10.1093/emph/eov009 (PMC4448095; doi:10.1093/emph/eov009)
Supplement: Supplementary Data [file supp_2015_1_123__index.html]

Adaptive learning can result in a failure to profit from good conditions: implications for understanding depression. — Adaptive learning can result in a failure to profit from good conditions: implications for understanding depression — Supplementary Data 

# Adaptive learning can result in a failure to profit from good conditions: implications for understanding depression

## Supplementary Data

files

**Files in this Data Supplement:**

- Supplementary Data - docx file
