# Supplementary material for: Adaptive learning can result in a failure to profit from good conditions: implications for understanding depression
Source: Evol Med Public Health. 2015 Apr 26;2015(1):123–35. doi: 10.1093/emph/eov009 (PMC4448095; doi:10.1093/emph/eov009)
Supplement: Supplementary Data [file supp_eov009_LearnedDepressionAPPENDIX_2015-02-20.docx]

**APPENDIX: Technical details of the model**

To allow optimal choices to be calculated after a given sequence of outcomes, we make use of bandit models (for theory see Gittins 1989, Robbins 1952; in the context of animal behaviour, see Krebs et al. 1978 and McNamara & Houston 1980).

Following standard theory, we assume there are 2 ‘arms’, A and B. At each time step, the individual pulls arm A or arm B. The outcome of pulling arm A at any time is known to be zero (on average); this corresponds to resting. Pulling arm B (trying) results in either a success (a positive pay-off, *X*) or a failure (a negative pay-off, *Z*). The probability of success on arm B, *p*_B_, is unknown; it takes one of two known values, *E*_g_ or *E*_b_ (for good and bad environments, respectively), and will sometimes change. The probability of *p*_B_ changing per time step is known to either be *α*_f_ or *α*_s_, where the switching probabilities *α*_f_ and *α*_s_ are known. The meta-probability of the switching probability changing (from *α*_f_ to *α*_s_ or vice-versa) in a given time step, *γ* = P(*α* switch), is also assumed to be known.

The sequence of decisions, rewards and changes to parameter values is shown in Figure A1.


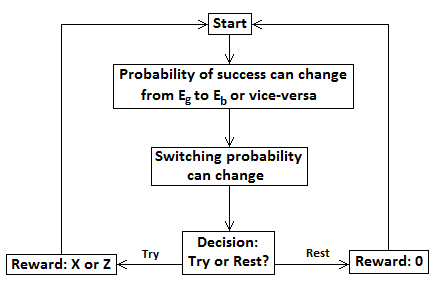


Figure A1: the sequential process of alterations and decisions.

The individual’s current belief about the probability of success and the switching rate on arm B is represented by the state variables P(*E*_i_, *α*_j_) for *i* = g, b and *j* = f, s (fast or slow switching). A strategy specifies the decision to rest (i.e. pull arm A) or try (i.e. pull arm B) for every combination of these state variables. We seek to identify the strategy that maximises the long-term rate of reward gain (henceforth the optimal strategy).

Although the belief state of an individual at any one time consists of four joint probabilities, P(*E*_i_, *α*_j_) for *i* = g, b and *j* = f, s, these are not independent as any one can be inferred from the other three (they must sum to 1). We therefore use a 3-dimensional grid of all possible (discretised) state variables. Each axis has 151 values (150 intervals between values).

The transition between belief states will depend on which action (try or rest) is taken from the current state, and, if trying, whether this resulted in success or failure. To choose the most appropriate action, each possibility is considered, with the action which results in the highest expected long-term rate of reward then being chosen. If the decision were made to rest, then the belief state is updated only in terms of the probabilities relating to the environment potentially having switched type: *α*_f_, *α*_s_ and *γ*. If the decision were made to try, then the belief state is updated both in terms of the environment potentially having switched type and according to the extra information resulting from success or failure.

**If resting**

We need to know how the P(*E*_i_, *α*_j_) distribution alters without a success or a failure. We assume that between decisions, the probability of success (*E*_g_ or *E*_b_) gets updated before the switching probability can change (*α*_f_ to *α*_s_ or vice-versa), as shown in Figure A1.

First consider the probability that *p*_B_ changes, from *E*_g_ to *E*_b_ or vice-versa:

P’(*E*_g_,*α*_f_) = (1 − *α*_f_)P(*E*_g_,*α*_f_) + *α*_f_ P(*E*_b_,*α*_f_)

P’(*E*_b_,*α*_f_) = (1 − *α*_f_)P(*E*_b_,*α*_f_) + *α*_f_ P(*E*_g_,*α*_f_)

P’(*E*_g_,*α*_s_) = (1 − *α*_s_)P(*E*_g_,*α*_s_) + *α*_s_ P(*E*_b_,*α*_s_)

P’(*E*_b_,*α*_s_) = (1 − *α*_s_)P(*E*_b_,*α*_s_) + *α*_s_ P(*E*_g_,*α*_s_).

Next we allow for *γ* changing the *α* values:

P’’(*E*_g_,*α*_f_) = (1 − *γ*)P’(*E*_g_,*α*_f_) + *γ* P’(*E*_g_,*α*_s_)

P’’(*E*_b_,*α*_f_) = (1 − *γ*)P’(*E*_b_,*α*_f_) + *γ* P’(*E*_b_,*α*_s_)

P’’(*E*_g_,*α*_s_) = (1 − *γ*)P’(*E*_g_,*α*_s_) + *γ* P’(*E*_g_,*α*_f_)

P’’(*E*_b_,*α*_s_) = (1 − *γ*)P’(*E*_b_,*α*_s_) + *γ* P’(*E*_b_,*α*_f_).

**If trying**

We also need to know how the P(*E*_i_, *α*_j_) values alter depending on success/failure. The calculations above show how possible changes in the environment affect belief state before obtaining additional data (from success or failure) when trying. This update is carried out using Bayes’ rule.

P(success) = P(*E*_g_,*α*_f_) P(success| start at *E*_g_,*α*_f_) + P(*E*_g_,*α*_s_) P(success| start at *E*_g_,*α*_s_)

+ P(*E*_b_,*α*_f_) P(success| start at *E*_b_,*α*_f_) + P(*E*_b_,*α*_s_) P(success| start at *E*_b_,*α*_s_)

= P(*E*_g_,*α*_f_) ((1 − *α*_f_) *E*_g_ + *α*_f_ *E*_b_) + P(*E*_g_,*α*_s_) ((1 − *α*_s_) *E*_g_ + *α*_s_ *E*_b_)

+ P(*E*_b_,*α*_f_) ((1 − *α*_f_) *E*_b_ + *α*_f_ *E*_g_) + P(*E*_b_,*α*_s_) ((1 − *α*_s_) *E*_b_ + *α*_s_ *E*_g_).

Bayes’ rule gives:

P’’(*E*_g_,*α*_f_|success) = P’’(*E*_g_,*α*_f_) P(success| start at *E*_g_,*α*_f_)/ P(success).

(Likewise for the other probabilities.)

We calculate the transition probabilities in a similar way relating to failure.

**State-dependent values and action selection**

Having calculated the transition probabilities relating to each possible action, the expected value of being at each point in probability space, P(*E*_i_, *α*_j_), is updated in turn. By ‘value’, here, we mean the difference between the expected long-term rate of reward from that position (given the strategy being assessed), and the expected long-term rate of reward averaged across all possible positions (see Puterman 1994). This is achieved iteratively by:

1. summing the expected reward (from the current position) in the coming time step and the expected value of the next location,
2. subtracting the value of a datum position (detailed below).

The value of each position is initialised to 0. By repeating the above steps for each point in probability space, the values gradually converge on the expected gain (or loss) with respect to the overall average gain per time step (Puterman 1994).

The expected value of resting, V(rest), at the current position, under iteration step 1 (above), is the sum of the expected immediate reward and the expected reward at the next position.

i.e., V(rest) = 0 + value at next position (4-dimensional position given by P’’).

Then the value of trying (under iteration step 1) is

V(try) = P(success) (*X* + value[P’’(*E*_g,_*α*_f_|success), P’’(*E*_b,_*α*_f_|success), P’’(*E*_g,_*α*_s_|success)])

+ P(failure) (*Z* + value[P’’(*E*_g_,*α*_f_|failure), P’’(*E*_b_,*α*_f_|failure), P’’(*E*_g_,*α*_s_|failure)]).

If V(try) > V(rest), then the value at that point is updated to V(try). Otherwise, the value at that point is updated to

p_forced_V(try) + (1 − p_forced_)V(rest)

where p_forced_ is the probability of forced attempts (i.e., being forced to try when the choice would have been to rest).

These calculations are carried out for each grid point in turn. Having subtracted the mean rate of gain (from each position), the values are then used as a new baseline from which to repeat the calculations; i.e., when the value of every grid point has been updated, each is reduced by the value corresponding to one (datum) position (that of the mid-point, (0.25, 0.25, 0.25, 0.25), where each of the 4 possibilities is equally likely), which allows the values to settle (as the system is ergodic). This iterative process is repeated until the values (and thus the decision matrix of whether it is best to try or not at a given grid position) have converged, at which point, we have a decision matrix which specifies, for any belief state, whether it is best to try or rest in the current time step.
